# PREMIUM: A French prospective multicenter observational study of factors impacting on efficacy and compliance to cetuximab treatment in first-line KRAS wild-type metastatic colorectal cancer

**DOI:** 10.1371/journal.pone.0243997

**Published:** 2020-12-21

**Authors:** L. Mineur, E. François, C. Plassot, J. M. Phelip, L. Miglianico, L. M. Dourthe, N. Bonichon, L. Moreau, R. Guimbaud, D. Smith, E. Achille, R. Hervé, J. M. Bons, S. Remy, R. Faroux, A. L. Villing, A. Mahamat, I. Rabbia, P. Soulié, I. Baumgaertner, N. Mathé, L. Vazquez, R. Boustany

**Affiliations:** 1 Institut Sainte-Catherine, Avignon, France; 2 Lacassagne Anticancer Center, Nice, France; 3 Institut Universitaire de Recherche Clinique, Montpellier, France; 4 Hopital universitaire CHU Nord Saint Etienne, Saint Etienne, France; 5 Saint Gregoire Hospital, Saint Gregoire, France; 6 Strasbourg Oncologie Libérale, Strasbourg, France; 7 Clinique TIVOLI, Bordeaux, France; 8 Clinique les Domes, Clermont-Ferrand, France; 9 CHU Rangueil, Toulouse, France; 10 Hopital Saint-André, Bordeaux, France; 11 Clinique de l’Orangerie, Strasbourg, France; 12 CH Privé Clairval, Marseille, France; 13 Polyclinique Saint-Francois, Desertine, France; 14 Centre d’Oncologie de la côte Basque, Bayonne, France; 15 CHD, La Roche-sur-Yon, France; 16 CH Auxerre, Auxerre, France; 17 CHU Archet II, Nice, France; 18 Cabinet médical, Orange, Paris, France; 19 CLCC Paul Papin, Angers, France; 20 CH Henri Mondor, Créteil, France; 21 Centre Clinique de Soyaux, Soyaux, France; McGill University, CANADA

## Abstract

**Background:**

Cetuximab improves progression-free survival (PFS) and overall survival (OS) in patients with KRAS wild type (wt) metastatic colorectal cancer (mCRC). Few data are available on factors impacting both efficacy and compliance to cetuximab treatment, which is, in combination with chemotherapy, a standard-of-care first-line treatment regimen for patients with KRAS wt mCRC.

**Patients and methods:**

PREMIUM is a prospective, French multicenter, observational study that recruited patients with KRAS wt mCRC scheduled to receive cetuximab, with or without first-line chemotherapy, as part of routine clinical practice, between October 28, 2009 and April 5, 2012 (ClinicalTrials.gov Identifier: NCT01756625). The main endpoints were the factors impacting on efficacy and compliance to cetuximab treatment. Predefined efficacy endpoints were PFS and safety.

**Results:**

A total of 493 patients were recruited by 94 physicians. Median follow-up was 12.9 months. Median progression-free survival was 11 months [9.6–12]. In univariate analyses, ECOG performance status (PS), smoking status, primary tumor location, number of metastatic organs, metastasis resectability, surgery, folliculitis, xerosis and paronychia maximum grade, and acne preventive treatment were statistically significant. In multivariate analysis (Hazard Ratios of multivariate stepwise Cox models), ECOG PS, surgery, xerosis and folliculitis were positive prognostics factors for longer PFS. Among all patients, 69 (14%) were non-compliant. In multivariate analysis, no variables were statistically significant. The safety profile of cetuximab was consistent with previous studies.

**Conclusions:**

ECOG PS <2, surgical treatment performed, and maximum grade xerosis or folliculitis developed were predictive factors of cetuximab efficacy on KRAS wt mCRC patients. Unfortunately, we failed in identifying predictive factors for compliance in these patients.

## Introduction

Colorectal cancer is the second most commonly reported cancer in females and the third most commonly reported cancer in males in France, averaging 43000 new cases and 17 000 deaths in 2018 [1]. Worldwide, one million new cases are estimated in 2018 and 881 000 deaths. Australia, North America and Europe have the highest incidence rate [1]. Over the last decade, the clinical outcome for patients with mCRC has improved greatly and physicians in Europe quickly integrated the KRAS status into the treatment strategy as early as 2008 [2]. This reflects the increase in the number of patients that are being managed by multidisciplinary teams and particularly a better strategic approach to systemic therapy delivery and development of ablative techniques procedures [3]. Although treatment decisions should be evidence-based, first-line management for (K)RAS wt mCRC patients remains being debated. Indeed, this choice remains very dependent on the disease presentation, e.g. dynamics of progression, extent of disease (liver/lungs or more), symptoms, patient comorbidities or mutations.

The addition of the anti-epidermal growth factor receptor (EGFR) monoclonal antibody cetuximab to the first-line chemotherapy improved clinical outcomes in the randomized phase III CRYSTAL trial, especially in patients with KRAS wt mCRC [4–6]. In CRYSTAL, for patients with KRAS wt mCRC, the cetuximab combination group presented a benefit in terms of PFS (median PFS, 9.9 months *vs* 8.7 months, HR = 0.68, 95%CI: 0.50–0.94), OS (median OS, 24.9 months *vs* 21.0 months, HR = 0.84, 95%CI: 0.64–0.1.11) and overall response rate (ORR) (59.3% vs 43.2%, OR = 1.91, 95%CI: 1.24–2.93). The CRYSTAL trial thus became a pivotal study in obtaining European Medicines Agency approval of the use of cetuximab as a first-line treatment for metastatic colorectal cancer [4].

This study provided robust evidence for clinical practice regarding cetuximab as a standard-of-care first-line treatment for patients with KRAS wt mCRC. However, controversy persisted based on limited data obtained from other trials (COIN and NORDIC VII), in which a lack of efficacy of cetuximab was observed [7, 8].

The possibility exists to request the marketing authorisation holder to conduct post-authorisation efficacy studies in order to complement available information from clinical trials by data collected in a larger and unselected population as sicker patients are often excluded from trials through eligibility criteria pertaining to comorbidity and performance status.

This study collects additional informations about side-effects, safety and benefits, and/or how well the medicine works when used widely. We considered it useful to evaluate the medical practices on the French territory, the progression-free survival (PFS) and to analyse the variables likely to influence it. These efficacy predictive factors are inherent in patients, disease, and side effects of cetuximab therapy.

Indeed, the predictive factors for PFS are important in current practice and guide physicians in the therapeutic strategy. Since these predictive factors may be related to the characteristics of the patients or their cancer, or even to the treatment type or these toxicities, the establishment of an observational study on therapy with cetuximab in current practice is more than relevant.

## Materials and methods

### Study design and patients

PREMIUM is a multicenter, prospective, observational study (NCT01756625) of French patients with KRAS wt mCRC who started on first-line cetuximab treatment from October 28, 2009 to April 5, 2012. Patient recruitment began on October 28, 2009 and follow-up ended on March 31, 2016. Study centers and investigators were chosen in order to be representative of the distribution of the care offer in the treatment of mCRC in France and according to the investigators practice in private centers or anti-cancer centers or university hospitals, to limit the biases related to the center effect.

Eligible patients were patients aged 18 years and above diagnosed with KRAS wt mCRC. All patients presenting with metastatic disease who had relapsed with or without adjuvant therapy and received cetuximab in the first-line treatment associated or not with chemotherapy (including 5FU and irinotecan or oxaliplatin) were included. Patients were excluded if they had received previous targeted therapy with bevacizumab or if they participated in a clinical trial. All patients had to provide written informed consent form (ICF) and the protocol was submitted to the competent regulatory authorities in France (the Committee for Data Processing in Health Research (CCTIRS) research minister as agreed with ethical committees). So, the case report forms (CRF) were reviewed and approved by the local Institutional Review Board, statistical department of the university of Nimes (BESPIM—Biostatistics, Clinical Epidemiology, Public Health, Medical Information).

### Study end points

Primary efficacy endpoints were PFS and PFS predictive factors. The event considered for progression-free survival was tumor progression, or death of any cause. Individuals who lost to follow-up of or did not present with a PFS event during the study were censored. The response to the treatment was evaluated according to RECIST v1.1 criteria.

A secondary objective concerned the identification of predictive factors for compliance.

These factors are inherent in the patient, the disease and the side-effects of the cetuximab treatment. Among factors inherent in the patients, we evaluated sex, age, sociodemographic data, ECOG Performance status, ethnicity, lifestyle and socio-professional category. Among the factors related to the disease, we evaluated TNM stage at diagnosis, primary tumor location, previous treatment, and resectability before or during the treatment. Finally, among the factors related to cetuximab treatment, we evaluated the administration frequency, the total dose received and the adverse events (AEs) of special interest (paronychia, xerosis, folliculitis). AEs were described and graded using the National Cancer Institute Common Terminology Criteria for Adverse Events (NCI-CTCAE) version 3.0.

### Procedure

During the inclusion period, any patient seen in consultation by a physician investigator and eligible based on the selection criteria was invited to participate. An information sheet was given to the patient, explaining the study objectives and the patient completed a data collection form. Patients were enrolled during an inclusion visit and then, patient data were prospectively collected at three months, six months, nine months, and twelve months after cetuximab initiation, at progression of disease or at 20 months after cetuximab initiation. The reasons for stopping treatment, whether definitively or temporarily, were listed. Only AEs of special interest were documented such as acne, paronychia, xerosis, and were assessed and graded according to NCI-CTCAE version 3.0.

Data collected during the study were retrieved from each center and captured using an electronic data system.

All patients were followed for up to 20 months or until progressive disease, from the time of enrolment. The median follow-up time was 12.9 months. No specific follow-up measures or evaluations were requested for this observational study. Evaluations for treatment outcomes were performed according to the current practice of individual investigators.

Cetuximab was administrated at an initial dose of 400mg/m^2^ followed by weekly doses of 250 mg/m^2^ or at 500 mg/m^2^ every 2 weeks and the choice of the chemotherapy regimen was at the physician’s discretion.

### Statistical analysis

Categorical variables were described using percentages while continuous variables were described using median values with the range or using mean values and standard deviations (SD). PFS was estimated using the Kaplan-Meier method. PFS was defined as the time from enrolment to the earlier of death or disease progression. The event considered for progression-free survival is tumor progression, or death of any cause and individuals who were lost to follow up or who did not present with a PFS event during the study were censored at the date when they were last known to be alive and free of disease progression.

Univariate analyses were performed for each covariable, using Kaplan -Meier methodology and log-rank test comparisons. Variables obtaining a significant log-rank test at the threshold of 15% (p value <0.15) in univariate analysis were then integrated into a multivariate Cox model. The so-called "stepwise" selection method was used, keeping only the significant variables at 5% (p value < 0.05). The Wald test was used. Multivariable stepwise Cox models were then fitted for final variable selection of prognostic factors on PFS. Hazard Ratios (HR) were presented as well as their 95% confidence intervals (CI).

As all the covariates are categorical variables, the proportional hazards assumption was the only one to test. It was performed for each covariate using the log (-log(S(t)) graphs. Deviance residuals are used to search for outliers and the Cox-Snell residuals were used to assess the goodness-of-fit of the Cox regression.

A similar method was used to evaluate predictive factors of compliance.

Statistical analysis was performed using SAS 9.3.

## Results

### Patients characteristics

**Fig 1:**
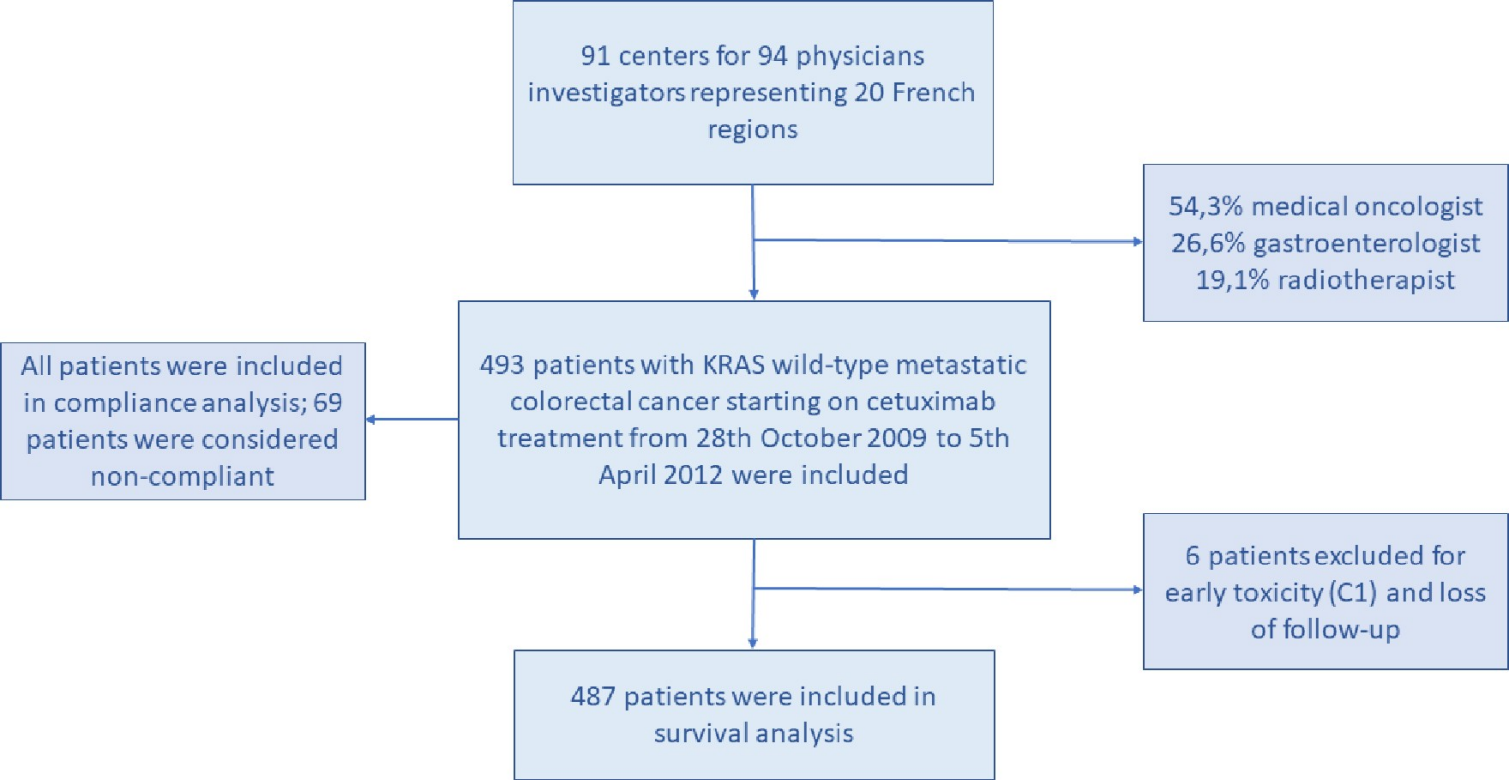
Trial profile.

A total of 493 patients who met the study criteria were enrolled but, because of stop due to early toxicities, only 487 patients could be considered in the survival analysis (Fig 1).

Oncologists and gastroenterologists have been selected and meet the following criteria: alls practicing in France in general hospital centers or university hospital centers (CHG, CHU) and/or private clinics and/or cancer centers (CLCC) that support patients with colorectal cancer.

Ninety-four physicians in ninety-one centers enrolled all the patients between October 2009 and April 2012. The demographic and clinical characteristics of patients are shown in Table 1. Percentages were calculated based on all enrolled patients (n = 493). The majority of patients were men (64%), of Caucasian origin (94%) and never smokers (57%). The ECOG performance status was 0–1 in 430 patients (88%). The median age was 66 years (29–88). Primary tumor location was in the left-sided colon in 35.5% (n = 175), in transverse colon in 5.3% (n = 26), in the right-sided colon in 21% (n = 103), in the rectosigmoid junction in 7.3% (n = 36), in the rectum in 31% (n = 152), and unknown in 1 patient. Overall, 331 patients (67%) had only one metastatic site (liver in 44%, lung in 5%, and other in 18%), and 86 patients (45%) had peritoneal carcinomatosis. In 70.4% of cases, the metastases were synchronous.

**Table 1:** Patients and treatment characteristics.

Population characteristics	n (%)	Median PFS (months)	p value
**Sex (n = 493; MD = 1)**			0.44
**Male**	314 (63.8)	11.7	
**Female**	178 (36.2)	9.6	
**Age, year (n = 493; MD = 0)**			0.85
**Median (range)**	66 (29–88)	10.3	
**<65 years**	229 (46.5)	11.7	
**65–74 years**	165 (33.5)	10.2	
**≥75 years**	93 (18.9)		
**Ethnicity (n = 493; MD = 12)**			0.38
**Caucasian**	452 (94)	11.3	
**Non-caucasian**	29 (6.0)	8.6	
**ECOG Performance status (n = 493; MD = 4)**			**<0.0001**
**0–1**	430 (87.9)	11.7	
**2–3**	59 (12.1)	6.0	
**Smoking history (n = 493; MD = 14)**			**0.0099**
**Never smoker**	275 (57.4)	10.0	
**Previous smoker**	157 (32.8)	13.9	
**Current smoker**	47 (9.8)	9.6	
**Social or familial isolation (n = 493; MD = 16)**			0.21
**Yes**	84 (17.6)	8.5	
**No**	393 (82.4)	11.7	
**Vocation activity (n = 493; MD = 10)**			0.59
**Unemployed/worker/employee**	128 (26.5)	11.8	
**Retired**	282 (58.4)	10.3	
**Profession average salary/artisan/liberal**	73 (15.1)	10.5	
**Education (n = 493; MD = 93)**			0.74
**Higher**	112 (28.0)	11.4	
**Lower**	288 (72.0)	10.4	
**Primary tumor location (n = 493; MD = 0)**			**0.15**
**Right colon**	103 (20.9)	10.4	
**Other**	390 (79.1)	11.4	
**Diagnostic M Tumor stage (n = 493; MD = 1)**			0.78
**M0**	174 (35.3)	11.0	
**M1**	318 (64.5)	11.3	
**Number of metastasis sites (n = 493; MD = 0)**			**0.0004**
**1**	331 (67.1)	12.0	
**>1**	162 (32.9)	9.6	
**Metastases resectability (n = 493, MD = 3)**			**<0.0001**
**Yes**	55 (11.2)	13.7	
**Potentially**	165 (33.7)	13.7	
**No**	270 (55.1)	9.7	
**Adjuvant chemotherapy (n = 487; MD = 0)**			0.25
**Yes**	125 (25.7)	8.6	
**No**	362 (74.3)	11.4	
**Surgery during treatment (n = 487; MD = 0)**			**<0.0001**
**Yes**	113 (23.2)	8.5	
**No**	374 (76.8)	NR	
**Number of metastases resections during treatment (n = 113; MD = 3)**			**0.066**
**≤ 2**	85 (77.3)	NR	
**≥ 3**	25 (22.7)	17.5	
**Metastasis delay (n = 493; MD = 0)**			0.47
**< 6 months**	347 (70.4)	11.3	
**6–12 months**	32 (6.5)	7.9	
**>12 months**	114 (23.1)	11.8	
**Paronychia toxicity grade (n = 487; MD = 8)**			**0.078**
**0–1**	406 (84.8)	10.6	
**2-3-4**	73 (15.2)	14.4	
**Xerosis toxicity grade (n = 487, MD = 8)**			**<0.0001**
**0–1**	333 (69.5)	9.7	
**2-3-4**	146 (30.5)	14.8	
**Folliculitis toxicity grade (n = 487; MD = 7)**			**0.0039**
**0–1**	279 (58.1)	8.7	
**2-3-4**	201 (41.9)	12.9	
**Hypomagnesemia (V2) (n = 487, MD = 100)**			0.23
**Yes**	38 (9.8)	7.7	
**No**	349 (90.2)	11.5	
**Acne preventive treatment (V2) (n = 487, MD = 1)**			**0.0007**
**Yes**	363 (74.7)	12.1	
**No**	123 (25.3)	9.8	
**Acne preventive treatment type (V2) (n = 363, MD = 0)**			0.65
**Tetracyclines**	229 (63.1)	11.7	
**Topical cream**	19 (5.2)	14.4	
**Tretracyclines + topical cream**	94 (25.9)	11.5	
**Other**	21 (5.8)	13.0	
**Acne curative treatment (V2) (n = 487; MD = 1)**			0.20
**Yes**	214 (44.0)	12.1	
**No**	272 (56.0)	9.8	
**Acne curative treatment type (V2) (n = 214; MD = 5)**			0.17
**Antibiotics**	155 (74.2)	11.5	
**Corticosteroids**	18 (8.6)	NR	
**Antibiotics+ corticosteroids**	12 (5.7)	11.7	
**Other**	24 (11.5)	12.6	
Chemotherapy protocol (V2) (n = 348; MD = 0)*			0.25
**Folfox**	129 (37.1)	9.6	
**Folfiri**	219 (62.9)	8.8	
Cetuximab administration frequency (V2) (n = 487; MD = 12)*			0.20
**Weekly**	100 (21.1)	11.8	
**Bimonthly**	375 (78.9)	10.3	
Received dose (V2) (n = 487; MD = 1)*			**<0.0001**
**< 2400 mg/m^2^**	140 (28.8)	6.5	
**≥ 2400 mg/m^2^**	346 (71.2)	12.0	
**RECIST response (V2) (n = 493; MD = 69)**	202 (47.6)	15.4	**0.0023 (responders vs stable)**
**Responders (Partial and complete)**			
**Stable**	137 (32.3)	13.0	
**Progressive**	85 (20.1)		

Abbreviations: **V2, Visit 2 at 3 months**; MD, Missing data; NR, Not Reached;

*Treatment-related variables not

included in multivariate analysis as non-randomized study

Among all patients, 11% (n = 55) presented with resectable metastasis, 34% (n = 165) presented with potentially resectable metastasis, and 55% (n = 270) with unresectable metastasis for the French investigators panel.

### Treatment

Most of the patients were started on cetuximab every two weeks (77%), while 20% of patients received cetuximab weekly (Table 1). For all visits combined, the median cumulative dose was 5000 mg/m^2^. Among the 493 patients, cetuximab could be maintained for more than 90 days in more than half of the patients (54%). The remaining 46% stopped cetuximab during the first 3 months, mostly because of progressive disease (18%), or because of a therapeutic break (15%).

Percentages for the occurrence of AEs were calculated considering all enrolled patients (n = 493) taking into account missing data (MD) for few toxicities in some patients. Most of the patients (n = 115, 74%) received prophylactic treatment to prevent skin reactions, it was tetracycline-based in 92% of the patients.

The three most common AEs observed were diarrhea (n = 247, 51%, DM = 5), anemia (n = 233, 48%, DM = 4), and nausea (n = 198, 41%, DM = 5). For folliculitis, xerosis and paronychia, for each patient the maximum grade across all visits was considered. More than two-thirds of patients developed folliculitis (n = 329, 68%, DM = 11), grade 3–4 for 55 patients (11%) and grade 2 for 30% of patients (n = 146). 55% (n = 264, DM = 11) of patient developed xerosis, grade 3–4 for 35 patients (7%), and grade 2 for 23% (n = 112). Paronychia was observed in 30% of patients (n = 142, DM = 12), grade 3–4 for 9 patients (2%), and grade 2 for 64 patients (13%).

Before enrolment, surgery was performed in 328 patients (67%), 278 for primary tumor resection (85%), 5 for metastasis resection (2%), and 45 for both (14%). 126 patients (26%) received adjuvant chemotherapy.

During the study, surgery was performed in 113 patients (23%), 22 (20%) in the context of primary tumor resection, 53 (47%) for metastases, 5 (4%) received radiofrequency ablation, 10 (9%) received radiofrequency ablation and surgery, and 23 (20%) another procedure (primary tumor surgery plus metastases surgery for 16 patients (70%)).

### Efficacy

**Fig 2:**
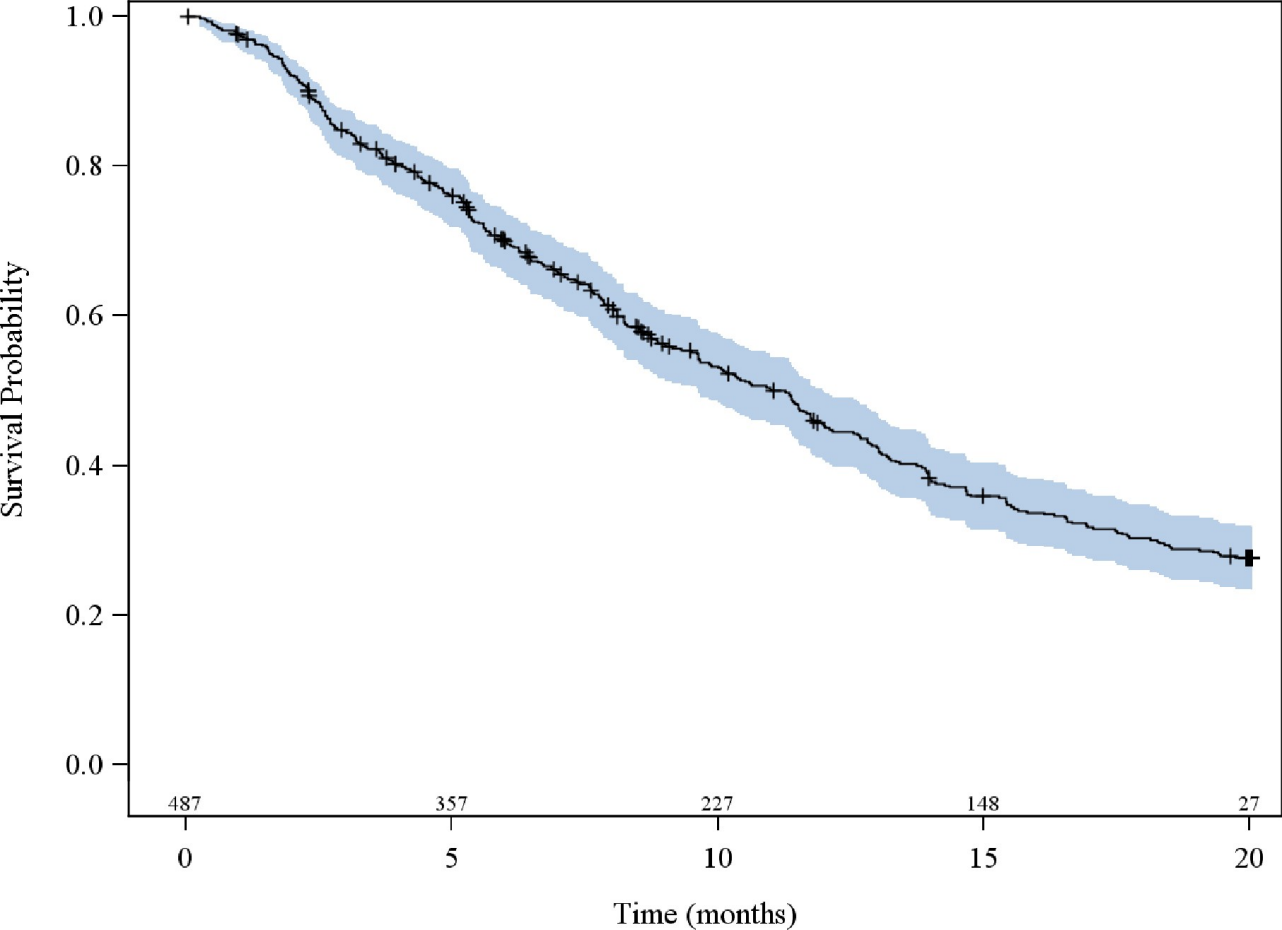
Kaplan-Meier plot for progression-free survival of mCRC patients on first-line cetuximab.

PFS analyses included all patients except 6 (n = 487), for whom survival data were unavailable. Overall median PFS was 11.0 [95%CI; 9.6;12.0] months with 324 (66.5%) patients having progressive disease at the time of analysis and the 20-months probability of progression-free survival was 28% [95% CI; 0.23–0.32] (Fig 2).

All visits combined, the treatment response could not be determined for 40 patients. Among the remaining 453 patients, considering the best response obtained, 52 (11.5%) had a complete response (radiological and metastatic disease resection), 197 (43.5%) had a partial response (decrease >30%), 114 (25.2%) had stable disease, and 90 (19.9%) had progressive disease. For responders’ patients at V2 (complete or partial response at 3 months evaluation), the median PFS was 15.4 months with a range from 13 to 19.7 months.

In this non-randomized study, we considered the type of chemotherapy used together with cetuximab, the cetuximab administration schedule, and the cumulative cetuximab dose received among the treatment-related variables, which not included in the multivariate analyses. Median PFS (95% CI) for the two most common first-line chemotherapy regimens administered with cetuximab was 8.8 months [7.6–11.0] for FOLFIRI, and 9.6 months [7.0–12.8] for FOLFOX. No statistically significant between-group differences were observed for PFS (p = 0.25). However, bivariate analysis of these two chemotherapy groups showed several significant differences (at the 5% threshold) including differences for conduct of surgery (p = 0.006), the rate of synchronous disease (p = 0.01), the resectability of metastases (p = 0.002), the cetuximab administration schedule (p = 0.0001), and the cumulative dose of cetuximab received (p = 0.002). It therefore appears difficult to compare these two treatments.

Median PFS (95% CI) for the two administration schedules regimens were weekly 11.8 months [9.8–14.7], and bimonthly 10.3 months [8.6–11.7]. No statistically significant between-group differences were observed for PFS (p = 0.20). Median PFS (95% CI) were 6.5 months [5.1–9.6] for cumulative cetuximab doses <2400mg/m2, and 12 months [10.6–13.8] for cumulative doses ≥2400 mg/m2. These PFS differences were statistically significant between-groups (p< 0.0001).

Cox regression analyses were used to evaluate the effect of patients’ characteristics on PFS (after selection in univariate analyses of variables to be included in the model). A baseline ECOG PS of 0–1 (95%CI; p = 0.0001), conduct of surgery during the treatment (95%CI; p<0.0001), or development of grade 2–4 xerosis (95%CI; p = 0.0022)or folliculitis (95%CI; p = 0.0059) appeared to be protective factors, significantly decreasing the likelihood of tumor progression (Table 2).

**Table 2:** Univariate and multivariate analyses of PFS according to patient characteristics.

Characteristics	n (%)	PFS (months)	Univariate analysis	Multivariate analysis
			p-value	p-value
**ECOG PS, (n = 489)**				
**0–1**	430 (87.9)	11.7	<0.0001	0.0001
**2–3**	59 (12.1)	6.0		
**Smoking history, (n = 479)**				
**Never smoker**	275 (57.4)	10.0	0.0099	
**Previous smoker**	157 (32.8)	13.9		
**Current smoker**	47 (9.8)	9.6		
**Primary tumor location, (n = 493)**				
**Right colon**	103 (20.9)	10.4	0.15	
**Other**	390 (79.1)	11.4		
**Metastases resectability, (n = 490)**				
**Yes**	220 (44.9)	13.7	<0.0001	
**No**	270 (55.1)	9.7		
**Surgery during the treatment, (n = 487)**				
**No**	374 (76.8)	NR	<0.0001	<0.0001
**Yes**	113 (23.2)	8.5		
**Folliculitis toxicity grade, (n = 480)**				
**0–1**	279 (58.1)	8.7		0.0059
**2-3-4**	201 (41.9)	12.9		
**Xerosis toxicity grade, (n = 479)**				
**0–1**	333 (69.5)	9.7	<0.0001	0.0022
**2-3-4**	146 (30.5)	14.8		
**Paronychia toxicity grade, (n = 479)**				
**0–1**	406 (84.8)	10.6	0.08	
**2-3-4**	73 (15.2)	14.4		
**Acne preventive treatment, (486)**				
**Yes**	363 (74.7)	11.8	0.0007	
**No**	123 (25.3)	7.6		

Abbreviations: NR, Not reached; HR, Hazard Ratio; CI, Confidence Interval; PFS, Progression-free survival; ECOG, Eastern Cooperative Oncology Group; PS, Performance status

A subgroup analysis for the treatment-response at V2 (3 months after enrolment) variable was performed, comparing PFS between responders (complete or partial response) and patients with stable disease. PFS was significantly better in patients with a response at V2 (95%CI; p = 0.002). Since this is a subgroup analysis, this variable could not be introduced in the Cox-regression.

Other subgroups analyses, especially in patients with synchronous disease (tumor size, lymph nodes disease, tumor location), in patients with cutaneous adverse events (preventive treatment type and curative treatment type), or in resected patients (number of resections) were performed and did not show any relationship with PFS.

### Compliance

Among the 493 patients, 69 (14%) were considered as non-compliant. Non-compliance to treatment was defined as patients who stopped cetuximab treatment for allergy, for skin toxicity (at the request of the patient or the physician agreement), by choice or by constraint of the mode of administration.

Table 3 shows the univariate analysis results, for each variable likely to influence treatment compliance. No covariate is significant at the 5% level. Only one covariate shows a trend (p<0.15); patients living with their families tend to be more compliant than patients living alone (p = 0.13). Since this covariate was the only one retained as a result of the univariate analysis, cox regression was not necessary.

**Table 3:** Compliance predictive factors: Univariate analysis.

		OR [95%CI]	p-value
**Physicians**			
**Practice place**		1.09 [0.6–1.99]	0.78
**Cancer center or University Hospital**	26 (27.7)		
**Other**	68 (72.3)		
**Speciality**		0.81[0.44–1.51]	0.51
**Gastroenterologist**	25 (26.6)		
**Oncologist**	69 (73.4)		
**Number of mCRC new cases supported/year**			0.64
**0–50**	30 (31.9)	1	
**51–75**	27 (28.7)	0.82[0.42–1.6]	
**>75**	36 (38.3)	0.73 [0.39–1.39]	
**Patient**			
**Sex**		1 [0.59–1.7]	0.99
**Male**	314 (63.8)		
**Female**	178 (36.2)		
**Age at diagnosic (years)**			0.24
**< 65**	229 (46.5)	1	
**65–74**	165 (33.5)	1.28 [0.71–2.3]	
**≥ 75**	93 (18.9)	1.74 [0.91–3.34]	
**Ethnicity**		0.45[0.1–1.94]	0.28
**Caucasian**	452 (94)		
**Non-caucasian**	29 (6)		
**Social or family isolation**		0.62 [0.33–1.15]	**0.13**
**Yes**	84 (17.6)		
**No**	393 (82.4)		
**Education**		1.05[0.56–1.96]	0.89
**Lower**	288 (72)		
**Higher**	112 (28)		

Abbreviations: OR, Odds Ratio; mCRC, metastatic colorectal cancer.

## Discussion

This prospective observational French multicenter study provided an analysis of the efficacy of and compliance with first-line mCRC cetuximab in combination, or not, with chemotherapy in a real-world setting in France. The observatory main objective was to assess PFS and related predictive factors in routine clinical practice. PFS was chosen as the primary endpoint based on the results of several published analyses, demonstrating that PFS can act as a surrogate for OS in the first-line treatment metastatic colorectal cancer [9, 10]. PFS is often chosen over OS in clinical trials as OS is impacted by the outcomes of subsequent therapeutic lines and does not directly evaluate the benefit of a given therapy [11].

The median PFS (11.0 months) of mCRC patients receiving first-line cetuximab in PREMIUM is similar to that reported in randomized control trials as CRYSTAL, OPUS, COIN, TAILOR and FIRE-3 trials (9.9 months, 8.3 months, 8.6 months, 9.2 months and 10 months respectively) [4, 7, 12–14], although it is slightly longer.

As expected, in PREMIUM a better baseline ECOG PS predicted longer PFS. Indeed, mCRC patients with ECOG PS≥2 experienced more treatment-related toxicities and had worse outcomes (mortality rate, response rate, PFs and OS) as demonstrated by Sargent et al. [15].

Similar to the CRYSTAL trial [4], the PREMIUM study highlighted a longer PFS in patients who developed substantial skin reactions. Indeed, a worse folliculitis toxicity grade (≥2) and a high xerosis toxicity grade (≥2) were statistically significant predictive factors for longer PFS. Different authors had already shown a positive correlation between a grade 2 or more cutaneous toxicity and patient survival [16, 17]. Skin reactions, resulting from the alteration of the mediation of epidermal basal keratinocytes by EGFR, seem to be a potential predictive biomarker to identify patients most likely to benefit from EGFR-inhibition. As different values of quality of life (EQ-5D health state index scores, health rating scores) and QLQ-C30 global health status scores did not change from baseline to safety follow-up, these scores appeared unaffected by the severity skin reactions [18, 19].

In the Cox regression analysis of PFS, differences in respect of another variable were found to be significant; the conduct of a metastatic disease surgery during the treatment was a statistically significant predictive factor for longer PFS. This finding is consistent with literature data, showing that primary tumor and/or metastases surgical resections extended patients survival compared with systemic treatment alone [20–23].

Interestingly, univariate analysis of PFS showed that having received a total cetuximab dose ≥2400mg/m^2^ during the first 3 months, even among the elderly, was associated with a longer PFS. However, this treatment-related variable could not be included in multivariate analyses as this is a non-randomized study.

Contrary to the findings by several studies [24–28, 37], the PREMIUM study did not find a significantly longer PFS among patients with a left-sided colon primary tumor (including rectal cancers) compared to another primary tumor location. We observed only a trend towards (p = 0.15) better PFS in patients with left-sided colon primary tumor location (in a univariate analysis). This outcome could be explained by other variables correlated with the primary tumor location. Indeed, left- and right-sided colon cancers exhibit distinctive clinical features and epidemiology, which could influence the differential oncologic outcomes [29]. Moreover, it should be noted that the influence of tumor BRAF mutation status was not investigated in the PREMIUM study whereas BRAF mutations negatively impact the treatment outcomes in mCRC [30, 31]. It has been shown that BRAF mutation is observed more frequently in right-sided colon cancer than in left-sided colon cancer, which might partly explain the higher response rate to cetuximab in patients with left-sided colon cancer observed in other studies [32, 33].

In the same way, NRAS and KRAS exons 3–4 mutations status were not investigated either. Note that a meta-analysis performed on mCRC suggests that patients with left-sided RAS wt tumours achieve a benefit from being treated with chemotherapy plus EGFR antibody therapy, while our study population received cetuximab-based treatment combined, or not, with chemotherapy [34].

A recent study by Lee et al. suggested that mutations in BRAF and NRAS, molecular subtypes and tumour methylation could provide an explanation for the association between survival outcome and primary tumor location. In this study, as in the PREMIUM study, after multivariate analysis, primary tumor location was not identified as a significant predictive factor of PFS and OS [35]. Other studies report a difference in cetuximab efficacy depending on the location of the primary tumor [24, 36, 37].

Finally, the absence of stratification according to tumour side, the heterogeneity of the treatments received by all the patients and the presence of some imbalances in the covariates between the two populations, were able to reveal some biases and could make the interpretation of these results hazardous, especially in the light of the knowledge that the underlying mechanisms for the differences in the survival outcomes between right-and left-sided colon cancer observed in all of these studies remain unclear.

The second aim of the PREMIUM study was to investigate factors predicting compliance in mCRC patients receiving first-line cetuximab treatment. We have suggested the hypothesis that many social forces push the patient towards poor compliance: level of education, social isolation or, at the opposite, patients with high social activity may not accept a specific skin toxicity of cetuximab. Based on our entire population, 14% of the 493 patients were considered as non-compliant. Univariate statistical analyses revealed a trend of a better compliance in patients living with family or with high social activity (p = 0.13; OR = 0.62[0.33–1.15]) than for those living alone. This finding seems logical, knowing the significance of family or social support in the care of cancer patients. No predictive factors of first-line cetuximab compliance in patients with mCRC was identified in our study.

This study has several limitations. Firstly, the benefits of anti-EGFR antibodies have been observed in all treatment lines as monotherapy and in combination with standard chemotherapy regimens and remain limited to mCRC with RAS wild-type genes. However, when the present study was performed, the mutation analysis was limited to exon 2 of the KRAS gene. Since then, extensive RAS gene analyses (including KRAS exon 3–4 mutations and NRAS exon 2–4 mutations) have been found to be more relevant than the exon 2 KRAS gene analysis alone. These additional so-called “minority” mutations are observed in about 10%-15% of additional patients with CRC. Its negative impact on our results remains low because only a small percentage of patients are affected. As well the observational design of this study and the frequency of assessments for survival data (every 3 months) could explain our slightly higher results in terms of PFS, which could supposedly have been overestimated. Not to mention the lack of independent central review radiological images across centers. Finally, these results are only from patients in care in French centers, limiting their extrapolation on an international scale.

This observational study confirms the outcomes seen with cetuximab in historical clinical trials and, thus, confirms the efficacy of cetuximab in first-line treatment for patients with RAS wt mCRC, extending PFS compared to systemic chemotherapy alone. However, the treatment response differs between patient subgroups. The PREMIUM study’s objective was to reveal predictive factors of PFS, that could be useful for current clinical practice. We demonstrated that ECOG PS <2, conduct of metastatic surgical treatment, and occurrence of maximum grade xerosis or folliculitis were predictive factors of cetuximab efficacy in KRAS wt mCRC patients. Unlike previous studies investigating anti-EGFR antibodies in mCRC but in accordance with Lee et al. [35], primary tumor location was not found to be a PFS predictive factor in our study. Further studies are needed to understand this outcome.
